# Anatomical and Functional Connectivity of Critical Deep Brain Structures and Their Potential Clinical Application in Brain Stimulation

**DOI:** 10.3390/jcm12134426

**Published:** 2023-06-30

**Authors:** Qiao Kong, Valeria Sacca, Meixuan Zhu, Amy Katherine Ursitti, Jian Kong

**Affiliations:** Department of Psychiatry, Massachusetts General Hospital, Harvard Medical School, Building 120, 2nd Ave., Charlestown, MA 02129, USA

**Keywords:** default mode network, non-invasive brain stimulation, subcortical structures, MRI, neuromodulation, scalp stimulation

## Abstract

Subcortical structures, such as the hippocampus, amygdala, and nucleus accumbens (NAcc), play crucial roles in human cognitive, memory, and emotional processing, chronic pain pathophysiology, and are implicated in various psychiatric and neurological diseases. Interventions modulating the activities of these deep brain structures hold promise for improving clinical outcomes. Recently, non-invasive brain stimulation (NIBS) has been applied to modulate brain activity and has demonstrated its potential for treating psychiatric and neurological disorders. However, modulating the above deep brain structures using NIBS may be challenging due to the nature of these stimulations. This study attempts to identify brain surface regions as source targets for NIBS to reach these deep brain structures by integrating functional magnetic resonance imaging (fMRI) and diffusion tensor imaging (DTI). We used resting-state functional connectivity (rsFC) and probabilistic tractography (PTG) analysis to identify brain surface stimulation targets that are functionally and structurally connected to the hippocampus, amygdala, and NAcc in 119 healthy participants. Our results showed that the medial prefrontal cortex (mPFC) is functionally and anatomically connected to all three subcortical regions, while the precuneus is connected to the hippocampus and amygdala. The mPFC and precuneus, two key hubs of the default mode network (DMN), as well as other cortical areas distributed at the prefrontal cortex and the parietal, temporal, and occipital lobes, were identified as potential locations for NIBS to modulate the function of these deep structures. The findings may provide new insights into the NIBS target selections for treating psychiatric and neurological disorders and chronic pain.

## 1. Introduction

The hippocampus, amygdala, and nucleus accumbens (NAcc) are critical deep brain structures involved in multiple functions, such as memory, emotional response, reward, learning and pathophysiology of chronic pain. Additionally, these brain regions are implicated in many psychiatric and neurological diseases; therefore, modulation of them may hold potential for treating such conditions.

For instance, the hippocampus, a part of the limbic system, is closely associated with Alzheimer’s disease (AD), Parkinson’s disease (PD), major depressive disorders (MDD), schizophrenia (SCZ), and epilepsy [1,2] due to its critical role in learning, memory, and high-level cognition [3].

The amygdala is another crucial limbic system structure associated with emotion and motivation, playing an essential role in processing both fear-inducing and rewarding environmental stimuli [4]. Aberrant activity/connectivity of the amygdala contributes to a wide range of disease states, including anxiety disorders, MDD, SCZ, bipolar disorder (BP), and autism spectrum disorder (ASD) [5,6].

The NAcc is a critical structure in modulating the processing of reward, pleasure, emotion, and motivation, as well as serving as a key limbic–motor interface [7]. Structural and functional abnormalities of the NAcc or its associated neural systems are involved in MDD, obsessive compulsive disorder (OCD), SCZ, Huntington’s disease (HD), PD, and chronic pain [8,9].

These structures do not work independently of each other but instead link to achieve various functions, such as long-term memory [10], goal-directed behaviors [11], and reward processing [12], making them potential neuromodulation targets for multiple disorders. Notably, these deep brain structures establish connections with common cortical areas, particularly the medial prefrontal cortex (mPFC), which is recognized for its pivotal role in cognitive and emotional processing, exerting top-down modulation of the limbic system [13,14]. Specifically, research has highlighted the involvement of the mPFC–hippocampus circuit in facilitating memory and learning processes [15,16]. The mPFC–amygdala circuit is important for social and emotional processing [17]. The NAcc serves as an integration hub for cortico-limbic information, coordinating adaptive motivated behavior by receiving executive control signals from the mPFC, conditioned associations and emotion from the amygdala, and contextual, spatial, and emotion-related inputs from the hippocampus [12,18,19].

Advances in neuromodulation technologies have enabled the use of invasive approaches, such as deep brain stimulation (DBS), and noninvasive brain stimulation (NIBS) interventions, such as transcranial electric stimulation (TES) and transcranial magnetic stimulation (TMS), to mitigate symptoms associated with psychiatric and neurological conditions [20,21]. The hippocampus, amygdala, and NAcc have recently been explored as effective DBS targets for treating epilepsy, OCD, SCZ, depression, and post-traumatic stress disorder (PTSD), among other diseases [22,23,24,25,26]. Despite the significant therapeutic benefit of DBS, the inherent surgery-related risks and complications from DBS have driven research toward less invasive alternatives [27].

Research indicates that both invasive and noninvasive stimulation of different brain regions can modify the same brain network to provide therapeutic benefits, which may be useful for translating therapy between neuromodulation modalities [28]. Moreover, researchers suggest that NIBS techniques can be applied through cortical targets to modulate deeper brain structures [29]. For example, TES with low intensities and small, high-definition electrodes over C3/C4 (10-10 EEG cap) was shown to generate an electric field in the hippocampus and amygdala of patients with drug-resistant epilepsy [30]. Thus, modulating the hippocampus, amygdala, and NAcc using NIBS may be a promising direction for treating brain disorders. Nevertheless, this could be challenging due to the localization of these deep brain structures and the accessibility of NIBS tools. The need for reliable montages to target these deep brain structures is thus essential for NIBS interventions.

One potential solution may be to identify accessible surface cortical regions that functionally and structurally connect with these subcortical brain structures. Studies have shown that TMS can modulate the neural activity in the hippocampus by stimulating the lateral parietal cortex, a superficial region that is functionally connected with the hippocampus [31,32]. Another study has shown that the clinical efficacy of different left dorsolateral prefrontal cortex (DLPFC) TMS sites for depression were related to intrinsic functional connectivity with remote regions (e.g., subgenual cingulate cortex) [33]. Moreover, investigators applied diffusion-imaging tractography to identify a superficial cortical target in the right frontal pole that displayed both anatomical and functional connectivity to the right Brodmann area 25 (BA25). Consequently, they found that TMS to the frontal pole resulted in a significant linear increase in blood-oxygen-level-dependent activation of BA25 with increasing TMS intensity [34]. Such findings suggest the potential of functional and structural connectivity in identifying surface brain stimulation targets. Therefore, we reasoned that we could indirectly modulate the function of these subcortical structures through direct stimulation of functionally and structurally related superficial cortical locations.

Seed-based resting-state functional connectivity (rsFC) analysis has been proposed as a primary approach for identifying connectivity-guided brain stimulation targets [35]. However, rsFC can vary over time in the same individual, affecting the reproducibility of brain stimulation target selection [36]. On the other hand, anatomical connectivity is temporally stable, and the underlying physical substrates allow signal propagation from the cortex to the subcortex [20]. Given the notion that structural differences to some extent dictate their roles in brain functions, diffusion MRI (dMRI) may thus complement or enhance fMRI for guiding target positioning [37].

Thus, this study combines resting-state fMRI and dMRI to identify brain surface stimulation targets that are structurally and functionally connected to three multifunctional subcortical structures (hippocampus, amygdala, and NAcc). The identified brain regions may be useful in identifying NIBS targets for neuropsychiatric disorders.

## 2. Materials and Methods

### 2.1. Participants

MRI data in this study were acquired from 119 healthy participants (age range: 18–60 years; 60 female) with no present reported major neurological or psychiatric conditions. The Partners Institutional Review Board (IRB) of Massachusetts General Hospital approved the study. All participants provided written informed consent before participating in the study.

### 2.2. MRI Data Acquisition

All MRI data were collected with a 32-channel head coil 3T Siemens (Skyra syngo) scanner at the Martinos Center for Biomedical Imaging. Resting-state functional (fMRI) data were obtained with an echo-planar imaging sequence using the following parameters: repetition time (TR): 3000 ms, echo time (TE): 30 ms, flip angle: 90°, slice thickness: 3 mm, voxel size: 3 × 3 × 3 mm^3^, 44 axial slices with 164 volumes acquired. Diffusion MRI data were collected with a single-shot spin echo EPI sequence in 60 gradient directions comprising 30 images with b: 600 s/mm^2^ and 30 images with b: 1200 s/mm^2^, and one b: 0 volume. The imaging parameters were: TR: 10,300 ms, TE: 85 ms, slice thickness: 2 mm, voxel size: 2 × 2 × 2 mm^3^, and 61 axial slices. T1-weighted images were acquired with a magnetization-prepared rapid gradient echo (MPRAGE) sequence with the following parameters: TR: 2500 ms, TE: 1.69 ms, flip angle: 7°, slice thickness: 1 mm, voxel size: 1 × 1 × 1 mm^3^, and 176 slices.

### 2.3. MRI Data Pre-Processing

Resting-state functional connectivity (rsFC) analysis was conducted using CONN toolbox version 21a (http://www.nitrc.org/projects/conn (accessed on 10 October 2022)). Preprocessing steps included the following steps: discarding of the first five volumes, slice-timing correction, realignment, outlier detection, indirect segmentation and normalization (MNI 152 template), smoothing with a Gaussian kernel of FWHM 6 mm, regression of nuisance covariates and head motion scrubbing, linear detrending, and filtering with a band-pass filter applied with a frequency window of 0.008–0.09 Hz.

The diffusion-weighted images were preprocessed using FMRIB Software Library, FSL version 6.0.3 (https://fsl.fmrib.ox.ac (accessed on 10 October 2022)). FMRIB’s Diffusion Toolbox (FDT) was used for head motion eddy current correction, and the images were affinely registered to the b0 reference image of each subject. Then, the DTI images were spatially normalized to the DTI data’s co-registered T1-weighted images.

### 2.4. Seed-Based Functional Connectivity Analysis for Target Generation

We selected the left and right hippocampus, amygdala, and NAcc as regions of interest (ROIs). All ROIs were defined using the Harvard–Oxford subcortical atlas (maxprob-thr25-2 mm). Functional connectivity analysis was computed between each ROI and each other voxel within the brain. In the first-level analysis, correlation maps were produced for each subject by extracting the time course of the BOLD signal from the left and right hippocampus, amygdala, and NAcc (respectively), and by computing Pearson’s correlation coefficients between the time courses in the ROIs and all other voxels in the whole brain. Correlation coefficients were transformed into Fisher’s z scores to increase normality.

Group-level analysis was performed on the functional connectivity maps (z values). A one-sample *t*-test was applied for each seed ROI to obtain a group-level correlation map (positive and negative correlation separately). For the whole-brain analysis, a voxel-level threshold at *p* < 0.001 and a cluster-level false discovery rate (FDR) *p* < 0.05 correction were applied.

As reported in previous studies [38,39,40], a brain surface mask was applied to constrain the results to the brain surface areas and further to identify cortically accessible stimulation sites. To optimize the stimulation targets generated by the rsFC, we used DPABI version 7.0 (http://rfmri.org/dpabi (accessed on 18 February 2023)) to increase T values by 0.5 based on the group-level correlation map (positive and negative correlation map separately), until 7~10 clusters with voxel values ranging from 30 to 800 were identified. Next, the peak MNI coordinates of these clusters were reported using the DPABI toolbox. The results were mapped onto an MNI standard template with the international 10–20 EEG system [41] using SurfIce (www.nitrc.org/projects/surfice/ (accessed on 10 October 2022)). The mapped locations were further checked visually by the investigators.

### 2.5. ROI-Based Probabilistic Tractography Analysis

To obtain the streamline density map from each ROI to the brain surface, bedpostX by FSL was first used to estimate the probability distribution of multiple fiber orientations in each voxel across the brain, under the default parameters (number of samples: 5000, curvature threshold: 0.2, step length: 0.5 mm, number of steps: 2000) [42]. Further, probabilistic tractography (PTG) was performed with probtrackx2 to compute a probabilistic tract from each voxel of one ROI to the brain surface, adopted as a waypoint mask. Only streamlines that passed through the waypoint mask would be considered valid. As reported in previous studies [37,43,44], the streamline density map of each ROI to the brain surface was binarized and thresholded of 1% for each subject. Finally, we combined individual streamline density maps into group maps. A threshold of 15% was set on the group-level probability map to remove those areas where only a few subjects showed connections [45].

### 2.6. Overlap of rsFC/PTG Analysis and Associations between Functional and Structural Connectivity within the Overlaps

Each ROI’s group streamline density map was binarized and applied to the corresponding group-level functional connectivity map (positive and negative rsFC map separately) to obtain overlapping brain regions. The overlaps were further restricted to the brain surface using the mask developed above. Next, following the methodology described in Section 2.4, we used DPABI toolbox to optimize these overlapping surface brain regions. Specifically, we increased T values by 0.5 based on the overlaps derived from the original positive/negative group-level correlation map and the group streamline density mask, until 7~10 clusters with voxel values ranging from 30 to 800 were identified. The peak MNI coordinates and final brain stimulation protocols were obtained using the DPABI and SurfIce toolbox.

We then explored the correlation between functional connectivity (FC) and structural connectivity (SC) within the overlapping brain regions. For the FC, we extracted each subject’s Fisher’s z-transformed mean value of each overlapping cluster, while the SC was extracted using two modalities: tract strength and fractional anisotropy (FA) along the white matter tracts. Both methods have been widely used to measure SC between the brain regions [46,47]. To obtain SC values, previously identified overlap regions were extracted and binarized for each ROI. Then, probtrackx2 was performed for each participant using the ROIs as seed masks, brain surface as a waypoint mask, and each overlapping region as a termination mask. The streamline density maps were obtained as described above. For obtaining the SC through tract strength, we calculated the mean value for all voxels within each streamline density map and divided it by the way total of streamline. Moreover, we also extracted the averaged FA values as a second method for the estimation of the SC.

The correlation between FC and SC (tract strength and mean FA) was estimated by Pearson’s correlation coefficient (*r*). Statistical significance was set at *p* < 0.05, and Bonferroni correction was applied for multiple comparisons. Statistical analysis was performed using JASP version 0.16.3 (http://www.jasp-stats.org (accessed on 8 March 2023)).

## 3. Results

### 3.1. The Left Hippocampus

#### 3.1.1. rsFC

**Figure 1:**
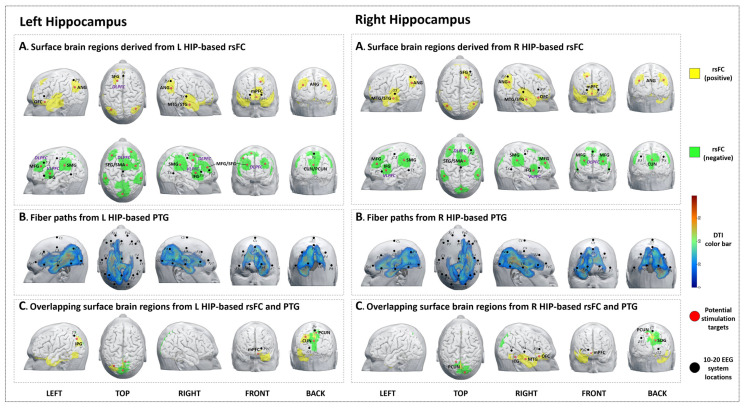
Potential brain stimulation targets of the left and right hippocampus. (**A-left**,**A-right**) Surface brain regions derived from L/R HIP-based rsFC. (**B-left**,**B-right**) Fiber paths from the L/R HIP-based PTG. (**C-left**,**C-right**) Overlapping surface brain regions derived from L/R HIP-based rsFC and PTG.

We identified 15 brain surface areas (6 positive and 9 negative) based on the rsFC results of the left hippocampus. The brain areas positively correlated with the left hippocampus were the left orbitofrontal cortex (OFC), DLPFC, right middle and superior temporal gyrus (MTG/STG), bilateral angular gyrus (ANG), and medial prefrontal cortex (mPFC). The brain areas negatively correlated with the left hippocampus were the bilateral DLPFC, ventrolateral prefrontal cortex (VLPFC), supramarginal gyrus (SMG), right supplementary motor area (SMA), left cuneus, and right precuneus (Table 1, Figure 1A-left).

#### 3.1.2. PTG

The PTG results showed that the white matter fibers connected the left hippocampus to brain surface areas that were mainly distributed at the left frontal and temporal lobes, as well as the bilateral parietal and occipital lobes (F3, Fz, P3, P4, O1, and O2 in the 10–20 EEG system) (Figure 1B-left).

#### 3.1.3. Overlap Regions between rsFC and PTG

The overlapping brain areas (2 positive and 2 negative) were the left inferior parietal gyrus (IPG), mPFC, bilateral cuneus, and the right precuneus (Table 1, Figure 1C-left).

### 3.2. The Right Hippocampus

#### 3.2.1. rsFC

We identified 15 brain surface areas (7 positive and 8 negative) based on the rsFC results of the right hippocampus. The brain areas positively correlated with the right hippocampus were bilateral MTG/STG, ANG, mPFC, right DLPFC, and OFC. The brain areas negatively correlated with the right hippocampus were bilateral DLPFC, VLPFC, SMG, cuneus, and right SMA (Table 2, Figure 1A-right).

#### 3.2.2. PTG

The PTG results showed that the white matter fibers connected the right hippocampus to brain surface areas that were mainly distributed at the right frontal and temporal lobes, as well as the bilateral parietal and occipital lobes (F4, Fz, T4, T6, P3, O1, and O2 in the 10–20 EEG system) (Figure 1B-right).

#### 3.2.3. Overlap Regions between rsFC and PTG

The overlapping brain areas (4 positive and 2 negative) were the right inferior temporal gyrus (ITG), MTG, OFC, mPFC, superior occipital gyrus (SOG), and left precuneus (Table 2, Figure 1C-right).

### 3.3. The Left Amygdala

#### 3.3.1. rsFC

**Figure 2:**
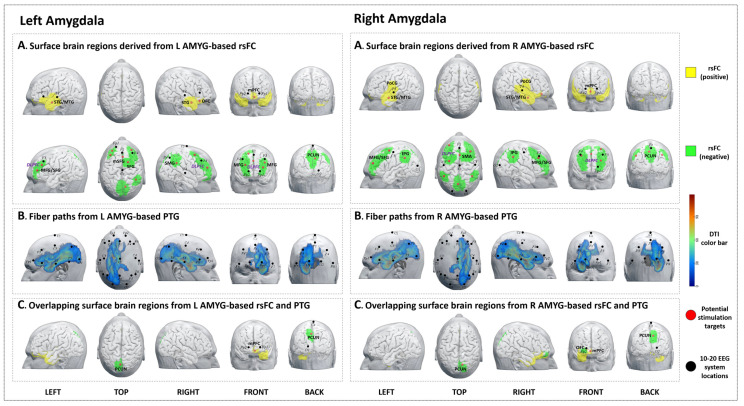
Potential brain stimulation targets of the left and right amygdala. (**A-left**,**A-right**) Surface brain regions derived from L/R AMYG-based rsFC. (**B-left**,**B-right**) Fiber paths from the L/R AMYG-based PTG. (**C-left**,**C-right**) Overlapping surface brain regions derived from L/R AMYG-based rsFC and PTG.

We identified 11 brain surface areas (4 positive and 7 negative) based on the rsFC analysis. The brain areas positively correlated with the left amygdala were the bilateral STG/MTG, mPFC, and right OFC. The brain areas negatively correlated with the left amygdala were the bilateral DLPFC, right SMG, and precuneus (Table 3, Figure 2A-left).

#### 3.3.2. PTG

The PTG results showed that the white matter fibers connected the left amygdala to brain surface areas that were mainly distributed at the left frontal, temporal, parietal, and occipital lobes (F3, Fz, C3, Cz, P3, Pz, and O1 in the 10–20 EEG system) (Figure 2B-left).

#### 3.3.3. Overlap Regions between rsFC and PTG

The overlapping regions from the left amygdala rsFC/PTG analysis (1 positive and 1 negative) were the bilateral mPFC and precuneus (Table 3, Figure 2C-left).

### 3.4. The Right Amygdala

#### 3.4.1. rsFC

We identified 10 brain surface areas (3 positive and 7 negative) based on the rsFC of the right amygdala. The brain areas positively correlated with the right amygdala were the bilateral mPFC, STG/MTG, and postcentral gyrus (PoCG). The brain areas negatively correlated with right amygdala were the bilateral DLPFC, IPG, SMA, and precuneus. (Table 4, Figure 2A-right).

#### 3.4.2. PTG

The PTG results showed that the white matter fibers connected the right amygdala to brain surface regions that were mainly distributed at the right frontal, temporal, parietal, and occipital lobes (Fp2, F4, Fz, C4, Cz, P4, Pz, and O2 in the 10–20 EEG system) (Figure 2B-right).

#### 3.4.3. Overlap Regions between rsFC and PTG

The overlapping brain regions from the right amygdala rsFC/PTG analysis (1 positive and 2 negative) were the right mPFC, OFC, and precuneus (Table 4, Figure 2C-right).

### 3.5. Left NAcc

#### 3.5.1. rsFC

**Figure 3:**
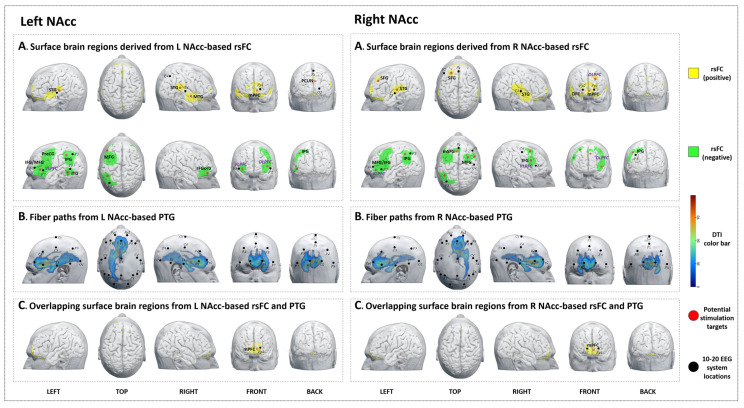
Potential brain stimulation targets of left and right nucleus accumbens. (**A-left**,**A-right**) Surface brain regions derived from L/R NAcc-based rsFC. (**B-left**,**B-right**) Fiber paths from the L/R NAcc-based PTG. (**C-left**,**C-right**) Overlapping surface brain regions from L/R NAcc-based rsFC and PTG.

We identified nine brain surface areas (5 positive and 4 negative) based on the left NAcc-rsFC results. The brain areas positively correlated with the left NAcc were the bilateral STG, right MTG, precuneus, and left mPFC. The brain areas negatively correlated with the left NAcc were the bilateral VLPFC, left DLPFC, PreCG, IPG, and ITG (Table 5, Figure 3A-left).

#### 3.5.2. PTG

The PTG results showed that the white matter fibers connected the left NAcc to brain surface areas that were mainly distributed at the bilateral frontal lobe and left temporal lobe (F3, F4, P3, Pz, and O1 in the 10–20 EEG system) (Figure 3B-left).

#### 3.5.3. Overlap Regions between rsFC and PTG

The overlapping brain region of the left NAcc rsFC/PTG analysis was the bilateral mPFC (Table 5, Figure 3C-left).

### 3.6. Right NAcc

#### 3.6.1. rsFC

We identified ten brain surface areas (5 positive and 5 negative) based on the right NAcc-rsFC results. The brain regions positively correlated with the right NAcc were the bilateral STG, mPFC, right OFC, and left SFG. The brain areas negatively correlated with the right NAcc were bilateral VLPFC, DLPFC, and left IPG (Table 6, Figure 3A-right).

#### 3.6.2. PTG

The PTG results showed that the white matter fibers connected the right NAcc to brain surface areas that were mainly distributed at the bilateral frontal lobe and right temporal lobe (Fp2, F4, Fz, and Cz based on the 10–20 EEG system) (Figure 3B-right).

#### 3.6.3. Overlap Regions between rsFC and PTG

The overlapping brain region of the right NAcc rsFC/PTG analysis was the bilateral mPFC (Table 6, Figure 3C-right).

## 4. Overlapping Surface Regions among Three Subcortical Structures Based on rsFC/PTG

**Figure 4:**
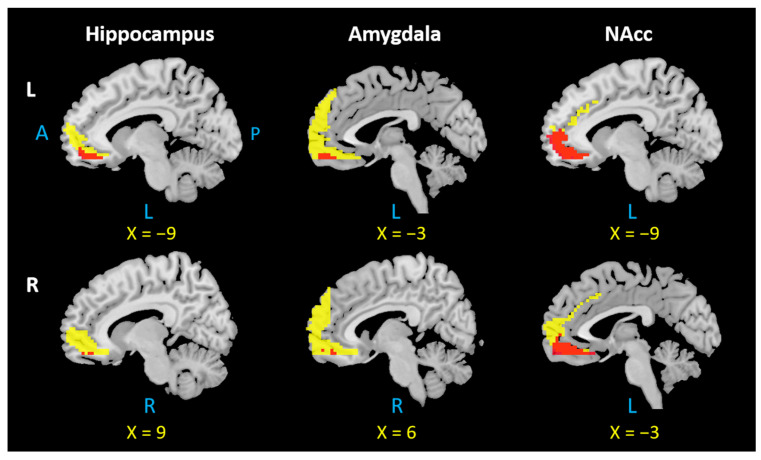
Overlap in the mPFC across all six seed regions. L: left, R: right, A: anterior, P: posterior. Notes: Each overlapping mPFC area was selected based on the positive rsFC and PTG results of the left and right hippocampus, amygdala, and NAcc. The mPFC region from positive rsFC results are indicated by yellow color; the overlapping mPFC region is indicated by red color.

**Figure 5:**
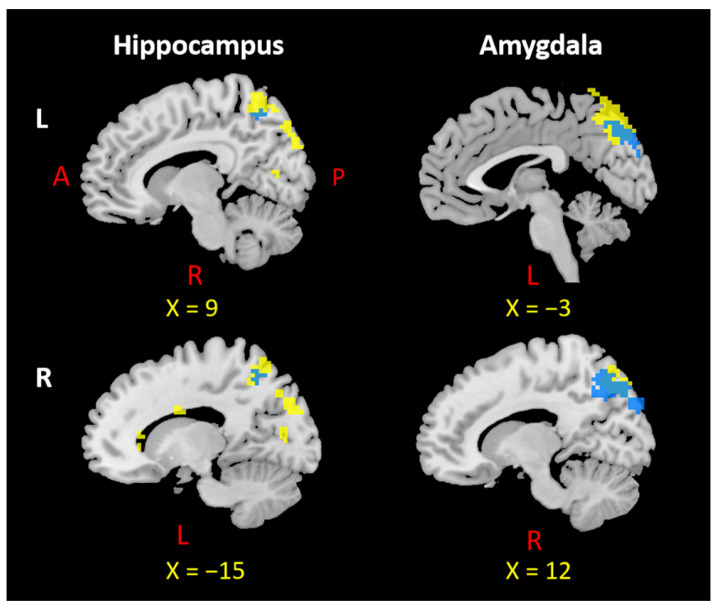
Overlap in the precuneus across four seed regions. L: left, R: right, A: anterior, P: posterior. Notes: Each overlapping precuneus area was selected based on the negative rsFC and PTG results of the left and right hippocampus and amygdala. The precuneus region from negative rsFC results appears in yellow; the overlapping precuneus region appears in blue.

We investigated the overlapping brain regions of the rsFC/PTG results across six ROIs. The results showed an overlap in the mPFC in all seed regions (Figure 4), while the precuneus was involved in the left and right amygdala and left and right hippocampus (Figure 5).

### Correlations between FC and SC in mPFC among Three Subcortical Structures

**Figure 6:**
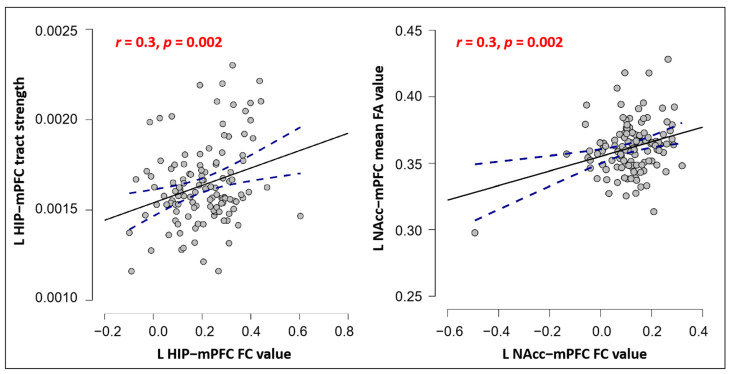
Pearson correlation scatterplot between FC and SC in hippocampus-mPFC (**left**) and NAcc-mPFC (**right**). Notes: *p* values survived the Bonferroni correction. The blue line indicates the 95% confidence intervals.

We further explored the associations of functional and structural connections in the overlapping mPFC areas of each ROI. The results showed: (1) a significant positive correlation between the tract strength and rsFC of the left hippocampus in the overlapping mPFC area (*r* = 0.3, *p* = 0.002, significant after Bonferroni’s correction, *p* < 0.05/12 = 0.004); and (2) a significant positive correlation between the FA and rsFC of the left NAcc in the overlapping mPFC area (*r* = 0.3, *p* = 0.002, corrected) (Figure 6). No other significant results were found.

## 5. Discussion

This study identified potential brain surface targets for NIBS of three deep brain structures (hippocampus, amygdala, and NAcc) by using rsFC and PTG methods on 119 healthy subjects. The results showed that the functional and structural connectivity of the three regions tend to be bilaterally distributed, while the ipsilateral connection is more robust than contralateral connectivity. Interestingly, we found that the mPFC is functionally and anatomically connected to all three subcortical regions, while the precuneus is connected to two regions (hippocampus and amygdala). The mPFC, precuneus, and other identified brain surface regions from rsFC analysis may be used as NIBS targets to influence the function of these subcortical regions and optimize treatment for psychiatric and neurological disorders in which these brain regions are involved. Furthermore, the identified brain surface areas may also be used as targets for other scalp stimulation methods, such as scalp acupuncture, which is based on brain anatomy and function [48]. In particular, the electrical scalp can be regarded as a new form of transcranial electrical stimulation (TES).

### 5.1. The mPFC Is Functionally and Anatomically Connected with the Hippocampus, Amygdala, and NAcc

We found that the mPFC is functionally (positively) and anatomically connected with the hippocampus, amygdala, and NAcc. The mPFC is a key node of the default mode network (DMN), which plays a crucial role in cognitive control and emotional regulation [49,50]. Disruption of the DMN is associated with a wide range of neurological and psychiatric diseases, including but not limited to AD, PD, SCZ, MDD, epilepsy, and attention deficit hyperactivity disorder (ADHD) [51].

Preliminary studies have provided evidence supporting the mPFC as a promising target for neuromodulation in the treatment of behavioral and emotional disorders, such as Huntington’s disease, obsessive compulsive disorder, and depression [52,53,54]. For example, a recent study found that tDCS applied to the mPFC can modulate subjective emotional experiences, accompanied by enhanced activation of the mPFC and other limbic regions, including the amygdala and ventral striatum. In the study, the tDCS electrode was placed vertically on the forehead, with side edges equidistant from the eyes and the bottom edge positioned 1 cm above the nasion [55]. In another study targeting the mPFC with the anode placed over Fpz (10–20 EEG system), investigators found that tDCS can modulate connectivity between the mPFC and subcortical reward circuits, particularly the right striatum, leading to improved safety learning in individuals with obsessive compulsive disorder [54]. These mPFC locations align with our own findings.

However, there is still much to learn about the underlying mechanism of its functioning when stimulated using NIBS. This may be complemented by work highlighting that hub nodes in the brain tend to have high average controllability in the DMN; therefore, modulation of hub regions can have high impacts on brain system functioning [56]. Our results support the idea that mPFC may be used as a key control node of DMN, thereby concurrently modulating activity in the hippocampus, amygdala, and NAcc to treat DMN-related diseases.

Studies have highlighted a primary role of mPFC–hippocampus circuits in memory retrieval and formation [57,58]. Research has suggested a correlation between stimulating this circuit and improved memory function after mind–body intervention [59]. Similarly, another study on rats revealed that stimulating mPFC could modulate hippocampal neuronal activity, which may lead to enhanced depression behaviors [60].

The mPFC has been recognized as a target for regulating mood and anxiety disorders through inhibiting brain areas involved in processing negative emotions, particularly the amygdala [61]. Recently, intensified transcranial direct current stimulation (tDCS) targeting the mPFC has been found to improve cognitive control, motivation, and emotional functions for social anxiety disorder by modulating the amygdala–frontal network [62]. Repetitive TMS (rTMS) of the mPFC has been found to facilitate emotional memories within an emotion–cognition network, including changes in hippocampus/amygdala-mPFC circuits [63]. Collectively, the mPFC may be a promising neuromodulation target to improve memory and affective disorders.

The NAcc and mPFC, two key components in the reward circuit, are thought to play critical roles in human social and affective function [64] and pain modulation [65,66]. Previous TES studies have indicated that stimulation of mPFC in patients and animal models induced antidepressant-like effects, which are associated with neural activation in NAcc [67]. A more recent study found that intracranial orbital mPFC signals can be used to predict spontaneous, chronic pain state in patients [68]. The mPFC-NAcc circuit may serve as a useful target in treatments for affective disorders and chronic pain.

Additionally, we observed a positive correlation between SC and FC in left hippocampus–mPFC as well as left NAcc-mPFC, which encompassed the mPFC (the overlapping region of each ROI), respectively. This finding aligns with the notion that functionally connected areas tend to be structurally connected, and that anatomical basis constrains FC to some degree [69,70]. Anatomical and functional connections between the mPFC and hippocampus are crucial for rapid learning and memory consolidation [16]. Furthermore, alterations in the SC-FC relations of mPFC–hippocampus/NAcc have been observed in several neuropsychiatric disorders, such as chronic pain, schizophrenia, and epilepsy [71,72,73]. This study demonstrated that FC and SC patterns within the mPFC–hippocampus/NAcc are interrelated in healthy individuals, and FC-SC coupling characteristics of these connections may act as potential neuromarkers for neuropsychiatric disorders.

Our results, along with those of previous studies, suggest that the mPFC is closely connected to the hippocampus, amygdala, and NAcc in both healthy and pathological states, offering potential for NIBS techniques to simultaneously modulate the activity of these three deep brain structures.

### 5.2. The Precuneus Is Functionally and Anatomically Connected with the Hippocampus and Amygdala

We found that the precuneus is functionally (negatively) and anatomically connected with the left and right hippocampus and amygdala. As another critical node of DMN, the precuneus is known as a remote interconnected node of the hippocampal intrinsic connectivity network [32]. Recently, a rTMS study has demonstrated the potential of targeting the precuneus as an effective intervention for subjective cognitive decline [74], associated with precuneus–hippocampus circuits, with the coil positioned at the Pz site (10–20 EEG system). Moreover, another rTMS study over the precuneus (the position based on individual T1-weighted MRI) has produced improvement on early memory symptoms of AD, and the clinical improvement is accompanied by modulation of the connectivity between the hippocampus and precuneus [75]. Our findings are consistent with these previous studies.

On the other hand, amygdala–precuneus FC has been associated with emotional regulation through attentional processes [76], and disrupted connectivity between them has been reported in psychiatric disorders such as MDD, BD, and PTSD [77,78,79]. Interestingly, we also observed positive functional connectivity between the precuneus and the left NAcc. Reduced NAcc–precuneus FC has been linked with neural factors of irritability related to ADHD [80], MDD, and Internet gaming disorder [81,82]. Nevertheless, few studies have used the precuneus as a NIBS target to treat such conditions.

Our findings, along with the above studies, endorsed the potential of targeting the precuneus using NIBS through the interaction between the precuneus and all three subcortical structures.

### 5.3. Other Hippocampus Functional Connectivity Analysis Results

We found intrinsic FC between the hippocampus and the frontal cortices (OFC/DLPFC/VLPFC), temporal, parietal, and occipital lobes. This expands on prior knowledge of the FC of the hippocampus [83], and highlights the potential of these areas as NIBS targets.

The OFC is associated with various brain functions, including memory and memory-related emotions, cognitive regulation, and reward [84,85]. Stimulating this area may help improve symptoms of cognitive decline and depression in various disorders [86,87,88]. The DLPFC, a vital node of the cognitive circuit involved in executive control and other critical cognitive processes, is a widely-used target for NIBS in treating various neurological and psychiatric conditions [89]. Moreover, the VLPFC, associated with memory and emotional functions, may be a suitable target for those with cognitive and emotional disorders [90]. Localizing appropriate NIBS sites for the DLPFC/VLPFC remains a challenge, and the findings of this study may contribute to the development of treatments for hippocampal-related diseases [91].

Previous work has demonstrated that NIBS of the lateral parietal cortex could alter hippocampal neural activity to improve memory function [83,92]. Our findings further support this NIBS implementation in treating memory disorders. We also found FC between the hippocampus and the temporal (STG/MTG/ITG) and occipital cortices (cuneus and SOG). The temporal lobe is involved in both semantic and episodic memory formation, while the interaction between the occipital lobe and hippocampus is essential for memory recognition and retrieval. These targets may be used to alleviate memory impairment in AD and semantic dementia cases [93].

Additionally, left–right hippocampus functional differences have been observed in both humans and animals, with the right hippocampus being involved in spatial memory, and the left in context-dependent episodic or autobiographical memory [94]. The left and right hippocampi also differ in their roles in short-term memory formation, with the right hippocampus facilitating it and the left suppressing it [95]. Therefore, their clinical relevance should be chosen flexibly and independently according to individual functions.

### 5.4. Other Amygdala Functional Connectivity Analysis Results

Our study revealed intrinsic FCs between the amygdala and OFC/mPFC/SMA/DLPFC, as well as the temporal and parietal lobes, which may be used as potential NIBS targets. This adds to prior knowledge of the FCs of the amygdala [96].

Regions of the prefrontal cortex, such as the OFC and mPFC, are known to be involved in regulating negative affect and fear responses [97]; therefore, targets in these regions may be suitable for treating mood disorders like depression and anxiety [98]. Furthermore, the SMA–amygdala circuit may account for the enhanced emotional arousal processing associated with PD, OCD, and irritable bowel syndrome [99,100,101], thus making the SMA a potential target for these conditions.

NIBS techniques, such as TMS and TBS delivered to the left DLPFC, have been approved by the U.S. Food and Drug Administration for treatment-resistant depression. Cole et al. proposed a rsFC-guided TBS protocol, which showed increased effectiveness compared to sham stimulation for treatment of depression [102]. The DLPFC target in the protocol was generated using rsFC with subgenual ACC as seed and DLPFC as a region of interest (around F3 of the 10–20 EEG system) [103]. This result is consistent with our findings.

Emotional attention modulation is thought to occur via projections from the amygdala to sensory processing areas, including the temporal cortex [104]. Additionally, the connections between the amygdala and PoCG integrate sensory information with emotional input [105], while the IPG perceives emotions in facial stimuli and interprets sensory information [106]. NIBS of the above areas hold promise for treating sensory and emotional disorders.

Although the FC results for the left and right amygdala were similar, they may have differential activation patterns in emotional processes. The left amygdala exhibited activation in cognitive and intentional control of mood, whereas the right amygdala is involved more in automatic emotion induction, relying less on explicit reflection processes [107].

### 5.5. Other NAcc Functional Connectivity Analysis Results

We found functional connections between NAcc and OFC/DLPFC/VLPFC, as well as the temporal and parietal lobes, which extend previous rsFC results of the NAcc [108]. These connections may have clinical applications for selecting NIBS targets.

Increased OFC-NAcc FC is associated with craving in alcohol use disorder and situational alcohol-seeking behavior [109]. TMS of the medial/lateral OFC may be effective in treating depression [110], making OFC-NAcc a promising option for treating substance use and mood disorders. The DLPFC/VLPFC-NAcc circuits are essential for emotional regulation. Anti-depression effects of TMS of the left DLPFC were found to be correlated with stimulated DLPFC-NAcc intrinsic FC strength [111]. Moreover, cognitive and neural evidence exists for targeting the VLPFC/DLPFC to enhance emotional regulation abilities [112], supporting our strategy of optimizing DLPFC/VLPFC targets based on NAcc rsFC.

Altered FC between the NAcc and the temporal area was implicated in temporal lobe epilepsy [113]. Variations in the NAcc and temporal lobes may be associated with PD and its impulse control disorders (ICDs) [114], with the severity of ICDs being correlated with IPG areas [115]. Targets located in the temporal gyrus and IPG could be useful for PD treatment.

While NIBS holds promise in targeting specific brain regions and modulating their activity, several considerations need to be acknowledged. First, like other interventions, there exist variability in individuals’ responses to stimulation, influenced by factors such as neuroanatomy, functional connectivity, and baseline brain state [116]. Moreover, variations in NIBS protocols, including intensity, duration, electrode position, and coil orientation, can significantly impact the effectiveness of stimulation [117]. Additionally, the precise mechanisms underlying NIBS effects remain unclear, and conflicting findings across studies further emphasizes the complexity of brain stimulation and the need for ongoing investigation [118]. Furthermore, it is essential to recognize the potential risks associated with NIBS techniques, such as unwanted side effects (e.g., seizures, headaches, or scalp discomfort), particularly when considering clinical applications in patient population [119]. Addressing these challenges involves optimizing stimulation protocols to account for individual variability, establishing standardized outcome measures, and enhancing reproducibility across laboratories. A comprehensive understanding of these limitations and challenges will foster responsible and effective utilization of NIBS techniques in future research and clinical applications.

## 6. Limitations

Our study has several limitations. First, the differential functions of left and right hippocampus, amygdala, and NAcc, and their associations with the corresponding diseases, remain unclear. For the convenience of discussion, we incorporated the connectivity associated with each side of the ROIs. To perform effective interventions for specific conditions, users may choose targets based on the specific neural circuit involved in each side of the structures. Secondly, it is important to note that our study was conducted on a sample of healthy subjects, which may limit the ability to fully capture the complexities and variations present in patients with psychiatric and neurological disorders. Further research is necessary to evaluate the efficacy and safety of targeting these brain regions in diverse clinical populations. Thirdly, our study relied on rsFC and PTG methods, both of which have inherent limitations and potential sources of bias. Factors such as the choice of seed regions, imaging parameters, and data analysis methods can impact the accuracy and reliability of these techniques in identifying and characterizing connectivity patterns between brain regions. Furthermore, our findings are based on group analysis, but individualized PTG and rsFC analysis may provide more accurate targets for brain stimulation tools (e.g., TMS). Nevertheless, for brain stimulation tools that do not have an accurate spatial resolution (e.g., TES), clinics that do not have MRI data available, or clinicians who do not have the expertise/resources to perform complicated brain imaging data analysis, our findings may provide valuable stimulation guidance. Finally, clinical trials and additional studies are needed to validate our findings.

## 7. Conclusions

We found that the mPFC, precuneus, and other surface brain regions may be used as potential brain stimulation targets to influence the activity/connectivity of the vital subcortical regions (hippocampus, amygdala, and NAcc). Our findings may shed light on identifying new NIBS targets for psychiatric and neurological diseases and chronic pain.

## Figures and Tables

**Table 1:** Surface brain regions derived from the left hippocampus resting-state functional and anatomical connectivity analysis.

rsFC	Cluster Size	Peak T	Peak Coordinate			Identified Brain Regions	10–20 EEG System Locations
			*x*	*y*	*z*		
Brain regions from resting-state functional connectivity analysis *							
Positive	66	14.60	−27	30	−15	L Orbitofrontal cortex	~2 cm inferior to F7
	181	13.66	−39	−69	33	L Angular	~3 cm inferior to P3
	44	10.69	−18	30	45	L Superior frontal gyrus	~3 cm left posterior to Fz
	194	13.07	45	−66	30	R Angular	~3 cm inferior to P4
	280	16.39	63	−6	−24	R Middle/Superior temporal gyrus	~3 cm anterior and inferior to T4
	571	20.42	−3	42	−12	Bil Medial prefrontal cortex	~0.5 cm inferior to the midpoint Fp2–Fp1
Negative	213	−7.38	−36	42	30	L Middle frontal gyrus	~1 cm anterior and inferior to F3
	76	−5.98	−54	12	6	L Inferior frontal gyrus	~2 cm posterior to F7
	237	−9.63	−57	−36	33	L Supramarginal gyrus	~midpoint to C3–T5
	221	−8.80	15	9	69	R Superior frontal gyrus/ Supplementary motor area	~2 cm right anterior to Cz
	435	−9.77	63	−36	36	R Supramarginal gyrus	~midpoint to C4–T6
	78	−6.85	45	6	51	R Middle frontal gyrus	~2 cm anterior and inferior to C4
	176	−7.99	57	15	9	R Inferior frontal gyrus	~2 cm posterior to F8
	354	−9.06	30	54	27	R Middle/superior frontal gyrus	~1 cm posterior and superior to Fp2
	431	−6.47	0	−90	27	L Cuneus/R Precuneus	~3 cm inferior to Pz
Overlapping brain regions from functional and anatomical connectivity analysis ^†^							
Positive	46	10.22	−30	−75	42	L Inferior parietal gyrus	~1 cm posterior and inferior to P3
	30	16.55	−9	42	−12	L Medial prefrontal cortex	~2 cm left inferior to Fp1
Negative	289	−6.47	0	−90	27	Bil Cuneus	~3 cm right superior to O1
	56	−5.70	9	−60	57	R Precuneus	~0.5 cm right inferior to Pz

* The threshold for positive rsFC is T > 8, and for negative rsFC is T < −4. ^†^ The threshold for positive rsFC is T > 4.5, and for negative rsFC is T < −3. Abbreviations: L: Left, R: Right; Bil: Bilateral.

**Table 2:** Surface brain regions derived from the right hippocampus resting-state functional and anatomical connectivity analysis.

rsFC	Cluster Size	Peak T	Peak Coordinate			Identified Brain Regions	10–20 EEG System Locations
			*x*	*y*	*z*		
Brain regions from resting state functional connectivity analysis *							
Positive	131	11.90	−60	−9	−18	L Middle/Superior temporal gyrus	~1 cm inferior to T3
	100	12.21	−42	−69	33	L Angular	~2 cm inferior to P3
	68	12.62	21	27	48	R Superior frontal gyrus	~2 cm left posterior to F4
	224	14.01	48	−63	36	R Angular	~2 cm anterior and inferior to P4
	694	20.39	63	−6	−21	R Middle/Superior temporal gyrus	~3 cm anterior and inferior to T4
	56	13.76	30	33	−15	R Orbitofrontal cortex	~3 cm inferior to F8
	512	20.52	3	33	−15	Bil Medial prefrontal cortex	~1 cm inferior to the midpoint Fp2–Fp1
Negative	215	−7.88	−36	48	24	L Middle frontal gyrus	~midpoint to Fz–F7
	134	−7.70	−45	18	3	L Inferior frontal gyrus	~1 cm posterior to F7
	260	−10.64	−54	−42	36	L Supramarginal gyrus	~0.5 cm posterior to the midpoint C3–T5
	174	−9.34	15	9	69	R Superior frontal gyrus/ Supplementary motor area	~junction of 1/3 and 2/3 Cz–F4
	275	−9.03	63	−39	36	R Supramarginal gyrus	~midpoint to C4–T6
	90	−7.63	54	15	3	R Inferior frontal gyrus	~2 cm posterior and inferior to F8
	212	−8.87	33	54	27	R Middle frontal gyrus	~midpoint to Fz–F8
	262	−6.92	3	−87	30	Bil Cuneus	~1 cm inferior to the midpoint of P3–P4
Overlapping brain regions from functional and anatomical connectivity analysis ^†^							
Positive	38	6.58	45	−45	−15	R Inferior temporal gyrus	~3 cm anterior and inferior to T6
	482	19.17	60	−12	−21	R Middle temporal gyrus	~3 cm anterior and inferior to T4
	51	12.61	36	33	−12	R Orbitofrontal cortex	~3 cm inferior to F8
	33	14.64	9	42	−15	R Medial prefrontal cortex	~1 cm right inferior to Fp2
Negative	30	−4.90	−15	−57	57	L Precuneus	~midpoint to Pz–P3
	407	−5.96	15	−93	33	R Superior occipital gyrus	~midpoint to Pz–O2

* The threshold for positive rsFC is T > 8.5, and for negative rsFC is T < −4.5. ^†^ The threshold for positive rsFC is T > 3, and for negative rsFC is T < −3.

**Table 3:** Surface brain regions derived from the left amygdala resting-state functional and anatomical connectivity analysis.

rsFC	Cluster Size	Peak T	Peak Coordinate			Identified Brain Regions	10–20 System Locations
			*x*	*y*	*z*		
Brain regions from resting state functional connectivity analysis *							
Positive	717	25.43	−27	3	−21	L Superior/Middle temporal gyrus	~3 cm inferior and anterior to T3
	414	15.67	27	6	−21	R Superior temporal gyrus	~3 cm inferior and anterior to T4
	36	11.49	33	33	−15	R Orbitofrontal cortex	~3 cm inferior to F8
	144	12.05	−3	42	−15	Bil Medial prefrontal cortex	~0.5 cm inferior to the midpoint of Fp1–Fp2
Negative	158	−6.02	−33	54	12	L Middle/Superior frontal gyrus	~1 cm right superior to Fp1
	46	−5.17	−39	39	30	L Middle frontal gyrus	~1 cm anterior and inferior to F3
	54	−7.19	3	33	36	Bil Medial superior frontal gyrus	~1 cm posterior to Fz
	156	−7.39	21	15	63	R Superior frontal gyrus	~midpoint to F4–Cz
	249	−8.18	48	−45	36	R Supramarginal gyrus	~midpoint to P4–T4
	658	−8.78	33	57	21	R Middle frontal gyrus	~midpoint to F4–Fp2
	732	−7.92	12	−69	45	R Precuneus	~midpoint to Cz–O2
Overlapping brain regions from functional and anatomical connectivity analysis ^†^							
Positive	66	11.29	−3	45	−15	Bil Medial prefrontal cortex	~1 cm inferior to the midpoint of Fp2–Fp1
Negative	211	−7.44	−3	−75	48	Bil Precuneus	~midpoint to Cz–O1

* The threshold for positive rsFC is T > 8, and for negative rsFC is T < −4. ^†^ The threshold for positive rsFC is T > 3, and for negative rsFC is T < −3.

**Table 4:** Surface brain regions derived from the right amygdala resting-state functional and anatomical connectivity analysis.

rsFC	Cluster Size	Peak T	Peak Coordinate			Identified Brain Regions	10–20 System Locations
			*x*	*y*	*z*		
Brain regions from resting state functional connectivity analysis *							
Positive	414	18.78	−24	3	−21	L Superior/Middle temporal gyrus/Postcentral gyrus	~3 cm inferior and anterior to T3
	783	31.06	27	3	−24	R Superior/Middle temporal gyrus/Postcentral gyrus	~3 cm inferior and anterior to T4
	148	13.34	3	48	−12	Bil Medial prefrontal cortex	~1 cm inferior to the midpoint of Fp2–Fp1
Negative	508	−7.72	−33	57	21	L Middle/Superior frontal gyrus	~midpoint to Fp1–F3
	52	−5.18	−33	3	63	L Middle/Superior frontal gyrus	~1 cm anterior and superior to C3
	187	−6.15	−51	−48	39	L Inferior parietal gyrus	~midpoint to C3–O1
	142	−7.75	3	24	45	Bil Supplementary motor area	~midpoint to Fz–Cz
	237	−8.34	45	−48	42	R Inferior parietal gyrus	~midpoint to C4–O2
	787	−8.83	39	36	39	R Middle/Superior frontal gyrus	~close to F4
	469	−8.81	−3	−75	54	Bil Precuneus	~1 cm left inferior to Pz
Overlapping brain regions from functional and anatomical connectivity analysis ^†^							
Positive	37	12.53	6	48	−12	R Medial prefrontal cortex	~1 cm right inferior to Fp2
Negative	36	−4.66	21	57	−3	R Orbitofrontal cortex	~close to Fp2
	216	−7.78	12	−72	45	R Precuneus	~midpoint to Cz–O2

* The threshold for positive FC is T > 7.5, and for negative rsFC is T < −4. ^†^ The threshold for positive rsFC is T > 3, and for negative rsFC is T < −3.

**Table 5:** Surface brain regions derived from the left NAcc resting-state functional and anatomical connectivity analysis.

rsFC	Cluster Size	Peak T	Peak Coordinate			Identified Brain Regions	10–20 System Locations
			*x*	*y*	*z*		
Brain regions from resting state functional connectivity analysis *							
Positive	108	8.54	−48	−21	6	L Superior temporal gyrus	~1 cm posterior and superior to T3
	60	7.28	60	−33	15	R Superior temporal gyrus	~2 cm posterior and superior to T4
	79	7.48	54	3	−12	R Middle temporal gyrus	~3 cm anterior and inferior to T4
	771	16.50	−9	42	−6	L Medial prefrontal cortex	~1 cm left inferior to Fp1
	297	8.16	9	−60	30	R Precuneus	~midpoint to Pz–O2
Negative	768	−7.21	−48	42	3	L Inferior/Middle frontal gyrus/Precentral gyrus	~1 cm anterior to F7, on the line F7–Fp1
	273	−6.07	−48	−45	51	L Inferior parietal gyrus	~2 cm anterior to P3
	31	−4.43	−57	−51	−6	L Inferior temporal gyrus	~1 cm anterior and inferior to T5
	273	−5.83	45	57	−9	R Inferior frontal gyrus	~1 cm anterior to F8
Overlapping brain regions from functional and anatomical connectivity analysis ^†^							
Positive	524	16.50	−9	42	−6	Bil Medial prefrontal cortex	~1 cm left anterior to Fp1
Negative	−	−	−	−	−	−	−

* The threshold for positive rsFC is T > 5, and for negative rsFC is T < 0. ^†^ The threshold for positive rsFC is T > 3, with no overlap between negative rsFC and PTG.

**Table 6:** Surface brain regions derived from the right NAcc resting state functional and anatomical connectivity analysis.

rsFC	Cluster Size	Peak T	Peak Coordinate			Identified Brain Regions	10–20 System Locations
			*x*	*y*	*z*		
Brain regions from resting state functional connectivity analysis *							
Positive	34	7.10	−24	42	39	L Superior frontal gyrus	~midpoint to F7–Fz
	147	8.77	−60	−21	9	L Superior temporal gyrus	~1 cm posterior and superior to T3
	383	7.59	57	−15	6	R Superior temporal gyrus	~close to T4
	56	8.18	24	33	−15	R Orbitofrontal cortex	~1 cm inferior to Fp2
	713	18.17	−3	45	−6	Bil Medial prefrontal cortex	~midpoint to Fp1–Fp2
Negative	409	−6.20	−48	51	−3	L Middle/Inferior frontal gyrus	~0.5 cm inferior to the midpoint of Fp1–F7
	147	−4.69	−48	−48	51	L Inferior parietal gyrus	~2 cm anterior to P3
	40	−5.41	−6	36	45	L Medial superior frontal gyrus	~1 cm left posterior to Fz
	31	−4.66	33	15	60	R Middle frontal gyrus	~midpoint to Fz–C4
	38	−4.63	57	18	30	R Inferior frontal gyrus	~midpoint to C4–F8
Overlapping brain regions from functional and anatomical connectivity analysis ^†^							
Positive	586	18.17	−3	45	−6	Bil Medial prefrontal cortex	~0.5 cm inferior to the midpoint of Fp2–Fp1
Negative	−	−	−	−	−	−	−

* The threshold for positive rsFC is T > 5.5, and for negative rsFC is T < 0. ^†^ The threshold for positive rsFC is T > 3, with no overlap between negative rsFC and PTG.

## Data Availability

The data that support the findings of this study are available from the corresponding author upon reasonable request.

## Abbreviations

NAcc: nucleus accumbens; NIBS: non-invasive brain stimulation; MRI: magnetic resonance imaging; rsFC: resting-state functional connectivity; PTG: probabilistic tractography; AD: Alzheimer’s disease; PD: Parkinson’s disease; MDD: major depressive disorders; TES: transcranial electric stimulation; TMS: transcranial magnetic stimulation; SCZ: schizophrenia; BP: bipolar disorder; ASD: autism spectrum disorder; OCD: obsessive compulsive disorder; HD: Huntington’s disease; MNI: Montreal Neurological Institute; ROI: regions of interest; FC: functional connectivity; SC: structural connectivity; FA: fractional anisotropy; mPFC: medial prefrontal cortex; OFC: orbitofrontal cortex; DLPFC: dorsolateral prefrontal gyrus; MTG: middle temporal gyrus; STG: superior temporal gyrus; ANG: angular gyrus; VLPFC: ventrolateral prefrontal cortex; IPG: inferior parietal gyrus; SMA: supplementary motor area; ITG: inferior temporal gyrus; SOG: superior occipital gyrus; PoCG: postcentral gyrus; tDCS: transcranial direct current stimulation; rTMS: repetitive transcranial magnetic stimulation; iTBS: intermittent theta pulse stimulation.
